# General practitioner residents’ mental health and satisfaction during their vocational training

**DOI:** 10.1186/s12875-026-03317-2

**Published:** 2026-04-14

**Authors:** András Mohos, László Kolozsvári, József Rinfel, Róbert Kiss-Kovács, Péter Torzsa, Gergely Ágoston

**Affiliations:** 1 Faculty of Medicine, Department of Family Medicine, University of Szeged, 6725 Tisza Lajos krt. 109, Szeged, Hungary; 2 Department of Family and Occupational Medicine, Faculty of Medicine, University of Debrecen, 4028 Kassai út 26, Debrecen, Hungary; 3 Department of Primary Health Care, University of Pécs, 7623 Rákóczi út 2., Pécs, Hungary; 4 Department of Family Medicine, Semmelweis University, 1091 Üllői út 25, Budapest, Hungary

**Keywords:** General practitioner, Family medicine, Vocational training, Mental health, Resident, Satisfaction

## Abstract

**Background:**

Primary care is the cornerstone of the healthcare system. In human resource recruitment, general practitioner resident vocational training plays a significant role. Satisfaction with the early years of a medical career influences future career plans and decisions. General practitioner residents’ mental health is also a key issue in the postgraduate training process. However, increasing workforce shortages and mental health problems among residents threaten the sustainability of primary care systems. The Hungarian primary care system faces a severe shortage of general practitioners. Therefore, it is crucial to know more about the career plans of resident doctors who have already entered family medicine training and to support them in remaining in this field.

**Methods:**

This was a national cross-sectional survey using a self-administered, paper-based questionnaire among general practitioner residents from all four Hungarian medical universities. Burnout, depression and hopelessness were assessed using validated instruments (Maslach Burnout Inventory, BDI-9, and Beck Hopelessness Scale). Data collection was carried out from December 2023 to January 2024. A total of 166 GP residents participated in our research, resulting in an overall response rate of 86%.

**Results:**

Reduced personal accomplishment was the most prevalent burnout dimension, affecting 40.7% of residents, while severe depression and hopelessness were present in 4.8% and 5.4%, respectively. Despite this, nearly 90% of respondents would choose family medicine again, and 71.9% were satisfied with their vocational training. Burnout, depression and hopelessness showed significant associations with lower satisfaction with career choice and vocational training. A significant majority plan to obtain an additional license or a specialization exam. Burnout, depression, and hopelessness showed significant correlations with satisfaction with career choice and vocational training.

**Conclusions:**

Most Hungarian general practitioner residents are satisfied with their career choice and vocational training. Targeted support for residents’ mental health and improvements in vocational training may contribute to retention in primary care and strengthen human resource recruitment.

**Supplementary Information:**

The online version contains supplementary material available at 10.1186/s12875-026-03317-2.

## Background

Primary care is the cornerstone of the healthcare system. It is crucial to have the right number and quality of healthcare professionals [1, 2]. However, many primary care systems in the world face increasing workforce shortages, threatening accessibility and continuity of care [3]. This shortage places increasing pressure on the training and retention of new general practitioners. Ensuring adequate human resource recruitment is a key issue to stop these negative trends. These findings highlight the importance of focusing not only on medical students, but also on residents who have already committed to family medicine training.

Hungarian health care system is relatively underfunded in the share of Gross Domestic Product (GDP), compared to the OECD average, and hospital centered. Primary healthcare services are provided in single-practice-based model by one general practitioner with one or two nurses. The mean practice size is about 1500 patients. General practitioners represented 12% (OECD average was 21%) of all doctors [4]. Based on human resource data the sustainability of the Hungarian primary health care system is under threat. In March 2026, 16% (1021/6373) of practices were vacant and the median age of the active GPs was 61 years [5].

In human resource recruitment, general practitioner residents’ vocational training plays a significant role, not only in acquiring the necessary skills but also in shaping personal experiences. Satisfaction with the early years of the medical career influences future career plans and decisions [6]. Family medicine residents’ satisfaction with their vocational training varies widely in the international literature. A common finding is the importance of relevant feedback and dedicated attention to their work. Improving basic skills and hospital practices is reported as the most problematic aspect of the training, while family practice training is evaluated positively [7, 8]. Residents are generally satisfied with their clinical competencies, while uncertainty and instability persist regarding management and practice leadership competencies [9]. Overall, vocational training quality and perceived preparedness appear to be key determinants of both career satisfaction and long-term retention in family medicine.

Postgraduate family medicine vocational training program lasts 36 months in Hungary. The first 2 years of the program providing clinical and primary care practices and theoretical knowledge, ends with a license exam. Specialization exam can be taken after 10 months of individual but supervised work by the GP-mentor in his or her practice and under supervision. In the last decade, approximately 7–9% (70–90 people) of graduating medical students have chosen family medicine [10]. Based on a previous Hungarian research only 5% of the medical students planned to choose family medicine as future career option [11].

In primary care settings a way of professional development to obtain additional license or specialization exam. In Hungary, at the time of the investigation, it was possible to obtain three types of licenses, which were introduced in 2014. The number of the issued licenses between 2014 and 2024: diabetology: 734 (in 2014: 494), hypertonology: 192 (in 2014: 136), palliative medicine: 145 (in 2014: 84). It was introduced a new license after data collection period, in 2026, Lifestyle medicine [10]. In 2016, 48% of the general practitioners had one (family medicine), 36% had two, 13% three, and 3% four specializations. Almost 20% of the respondents reported internal medicine, anaesthesiology was described in more than 2%, while pulmonology, surgery, gastroenterology, obstetrics and gynecology and diabetology also reached almost 1%. Occupational medicine as a part time job reported by about 1500 general practitioners in Hungary [12].

Burnout is classified by the WHO as an occupational phenomenon related to stress and not a medical condition, and is defined as “a particular response to stress involving psychological withdrawal. It is a costly coping mechanism seen as the endpoint of a process, which begins with the stress response” [13]. International studies show that this conditions is highly prevalent among general practitioners and residents. Burnout is not only the healthcare professionals’ personal problem, but also negatively affect doctors’ satisfaction, workload and associated with reduced quality of care, increased medical errors and early career attrition [14–17]. General practitioners are particularly affected by physician burnout, although absolute levels of burnout vary from study to study. In the multi-centered EGPRN study 43% of respondents scored high for emotional exhaustion, 35% for depersonalization and 32% for personal accomplishment [18]. In a 2022 review the pooled burnout rate showed 37%, 28%, and 26% of general practitioners suffered from high emotional exhaustion, high depersonalization, and low personal accomplishment, respectively [19]. Among German GP residents high level of emotional exhaustion was found in 33.5%, high level depersonalization in 35.2% and high level reduced personal accomplishment in 14.6% [15].

Depression is a common and serious mood disorder with heterogeneous symptoms, which can affect not only the patients’ mood but also their daily activities, social interactions, and somatic health. It can be diagnosed based on the DSM-V criteria [20]. According to studies from the last two decades, 7.1- 8% of the Hungarian population was affected by depressive symptoms during a one year period, the lifetime prevalence was 15.1–18% and point prevalence was 2,6 − 5% [21–25]. It was found moderate or severe depression symptoms in 10.9% of 211 GP trainee in Germany which was higher than in the general population [15]. A systematic review showed an overall pooled prevalence of depressive symptoms around 28.8%, ranging from 20.9% to 43.2% among resident physicians [26].

Hopelessness has many conceptualizations, but common elements include negative expectations or pessimistic attitudes toward the future or self, as well as the overestimation of adverse events or underestimation of positive outcomes. Hopelessness is an independent and strong predictor of suicide risk [27–29]. In the Hungarostudy 2002 population high suicide risk (Beck hopelessness score ≥ 6) was identified for 8.66% of the respondents [25]. Laramée et al. found that the rate of suicidal ideation with a plan during residency was 18.1%, and the rate of suicide attempt during residency was 2.9% among Canadian GP residents [30].

Because of special working conditions and the high number of doctor-patient interactions, burnout and mental health issues are prevalent among general practitioners and GP residents as well and have to be a key point in the postgraduate training process [31, 32]. Burnout, depression, and hopelessness decrease patient satisfaction and the quality of care, including an increased number of medical errors and negatively impact doctors’ well-being [33–35]. These factors can negatively affect residents’ study skills, academic attainment, training outcomes, quality of life, and may lead to doctors’ exit from primary care and a career change [17, 36]. Rurik et al. found that work-related stress and overload due to unnecessary administrative tasks were mentioned as the most important risk factors for burnout among Hungarian family physicians [37].

In the last decade, several studies on Hungarian residents’ mental health, working conditions, and willingness to work abroad were conducted, but there is limited recent information related to GP residents [38, 39]. However, recent data focusing specifically on general practitioner residents are scarce, despite their critical role in sustaining primary care services. Therefore, this national study aimed to assess the prevalence of burnout, depression and hopelessness among Hungarian GP residents, and to explore their associations with vocational training satisfaction and future career plans.

## Methods

### Study design and participants

This was a national cross-sectional survey using a self-administered, paper-based questionnaire among general practitioner residents. Participation was anonymous and voluntary. During routine resident meetings, residents received the informed consent form and the questionnaire, completed it, and handed it in before leaving. Participation was voluntary, anonymous, and no incentives were provided. Paper-based data collection during resident meetings was chosen to maximize response rates. Data collection was carried out from December 2023 to January 2024.

### Data collection

Sociodemographic data, including gender, age, and place of origin were collected. (Categorisation of the settlements: capital city: Budapest; big city: population is higher than 20000; small town: population is lower than 20000 and if, to the knowledge of the respondent, the settlement is a town; village: if, to the knowledge of the respondent, the settlement is a village.) Questions also addressed satisfaction with specialty choice and vocational training. Burnout was assessed using the Maslach Burnout Inventory (MBI). We classified physicians into three groups based on the perceived level of burnout (high, intermediate, low) based on cut-off values reported by Maslach et al. [40].

Depression was measured using the shortened Beck Depression Inventory (BDI-9). BDI consists 9 items. Each item is rated on a 4-point scale from 0 to 3. 0–not typical; 1–rarely typical; 2–typical; 3–very typical. The total score has to be multiplied by 2.33. 0–9 points: No depression symptoms, 10–18: mild depression symptoms, 19–24: moderate depression symptoms, 25-: severe depression symptoms. BDI as a screening tool for depression in primary health care patients with a cut-off score of 19 was found to have a 68.6% sensitivity and 97.5% specificity by Rózsa et al. [41].

Hopelessness was measured with the short − 4-item - Beck Hopelessness Scale. Answers were evaluated on a four-point Likert scale ranging from 0 to 3 (0–not typical; 1–rarely typical; 2–typical; 3–very typical). 0–2 points: No hopelessness symptoms, 3–5 points: Mild hopelessness symptoms, 6- points: severe hopelessness symptoms [25]. The questionnaire was pilot-tested for clarity and comprehensibility by a group of GP residents at the Department of Family Medicine in Szeged and no changes have been made. [Supplement 1.]

### Data analysis

There was not significant differences between the results of the universities; therefore, the results were analyzed together. Descriptive statistics were presented as counts and percentages for categorical variables, and as means and standard deviations (SD), complemented by medians and quartiles where appropriate, for continuous variables. Group differences between categorical variables were analyzed using χ² tests, while differences between continuous variables were examined using one-way analysis of variance (ANOVA), followed by Scheffé post hoc tests when applicable. Spearman rank correlation was used to measure correlation between to ranked data. IBM SPSS Statistics 24 software was used for statistical analysis. Sample sizes varied due to missing data, which were handled by pairwise deletion. Statistical significance was set at *p* < 0.05 with a 95% confidence level (CI: 95%). In the case of multiple comparisons, as five factors (emotional exhaustion, depersonalisation, reduced personal accomplishment, depression, and hopelessness) were examined, the level of statistical significance was set at p/5 (i.e. *p* < 0.01) in order to avoid an increased risk of a type 1 error.

### Ethics statement

The study was approved by the Medical Research Council, Hungary (reference number BM/25034-3/2024). Participation was voluntary, and informed consent was obtained from all participants prior to data collection. Data were collected anonymously and processed in accordance with applicable data protection regulations (GDPR). Participants were informed of their right to withdraw at any time without any consequences. The study was conducted in accordance with the Declaration of Helsinki.

## Results

A total of 193 general practice (GP) residents were invited to participate in the study. Of these, 166 completed the questionnaire, resulting in a response rate of 86%. which is considered high for survey-based research.

The mean age of the participants was 31.9 ± 7.04 years, and 65.8% of respondents were female. We can assume that the characteristics of the participant group of residents and the non-participant group of residents are not different. The gender distribution was similar among non-respondents, suggesting no substantial difference between respondents (106/161: 62.1% female) and non-respondents (18/27: 66.7% female) in this respect. Detailed sociodemographic characteristics are presented in Table 1.

**Table 1:** Sample characteristics

Variable	Valid (*N*)	*N* (%)
Age [mean ± SD]	164	31.9 +- 7.04 years
Women	161	106 (65.8)
Marital status	166	
Single		23 (13.9)
In a relationship		57 (34.3)
Married		80 (48.2)
Divorced		4 (2.4)
Widowed		1 (0.6)
Comes from…	162	
the capital city		30 (18.5)
a big city		67 (41.4)
a small town		36 (22.2)
a rural area		29 (17.9)
Lives in…	163	
the capital city		51 (31.3)
a big city		82 (50.3)
a small town		19 (11.7)
a rural area		11 (6.7)
Workplace	164	
the capital city		49 (29.9)
a big city		66 (40.2)
a small town		27 (16.5)
a rural area		22 (13.4)
Family medicine as career	164	
First choice		90 (54.9)
Not first choice		74 (45.1)

**Fig. 1:**
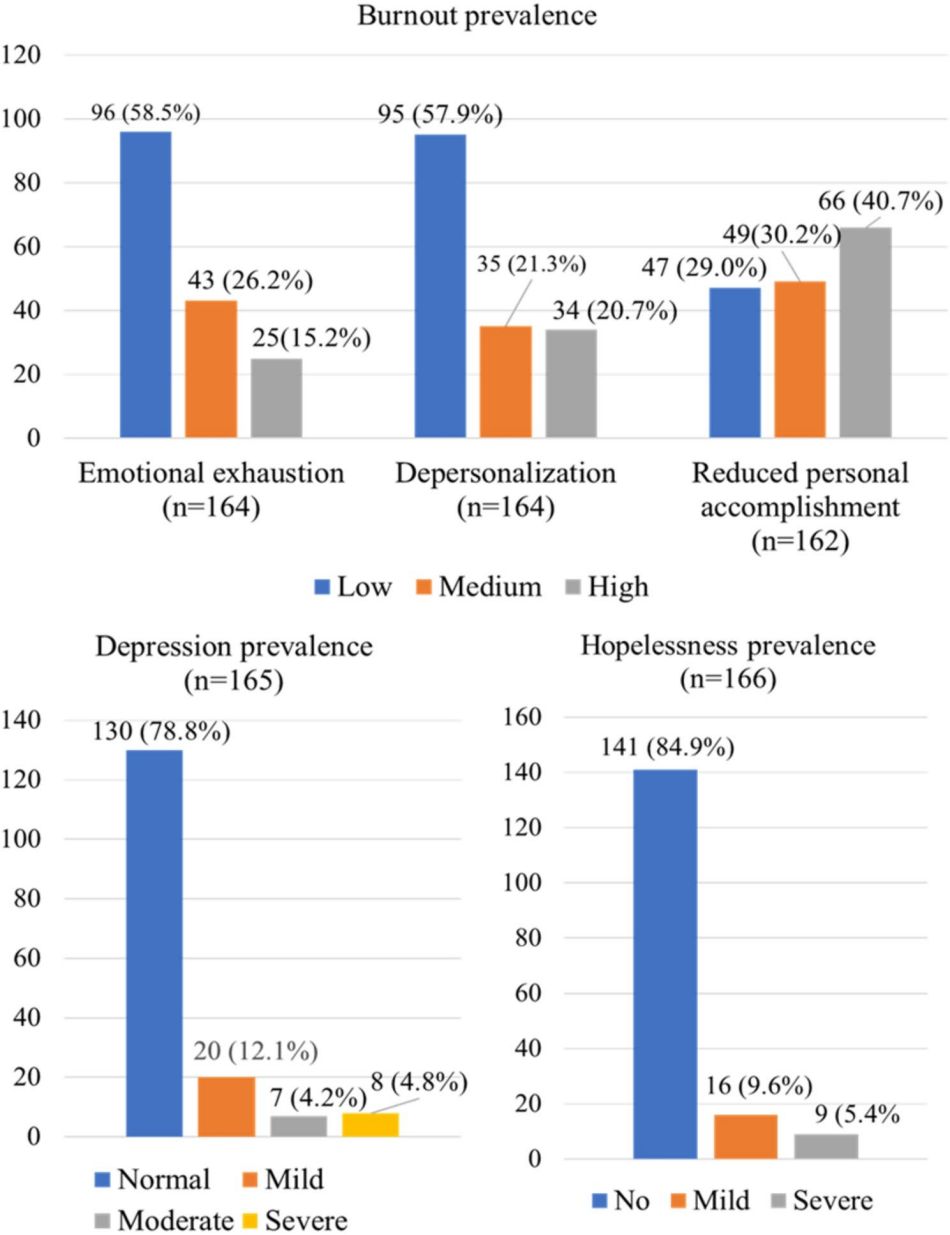
Burnout, depression and hopelessness among resident doctors

High-level reduced personal accomplishment was the most prevalent burnout dimension, affecting 40.7% of residents, compared with high levels of emotional exhaustion (15.2%) and depersonalization (20.7%). Severe depression was identified in 4.8% and severe hopelessness in 5.4% of respondents (Fig. 1).

**Fig. 2:**
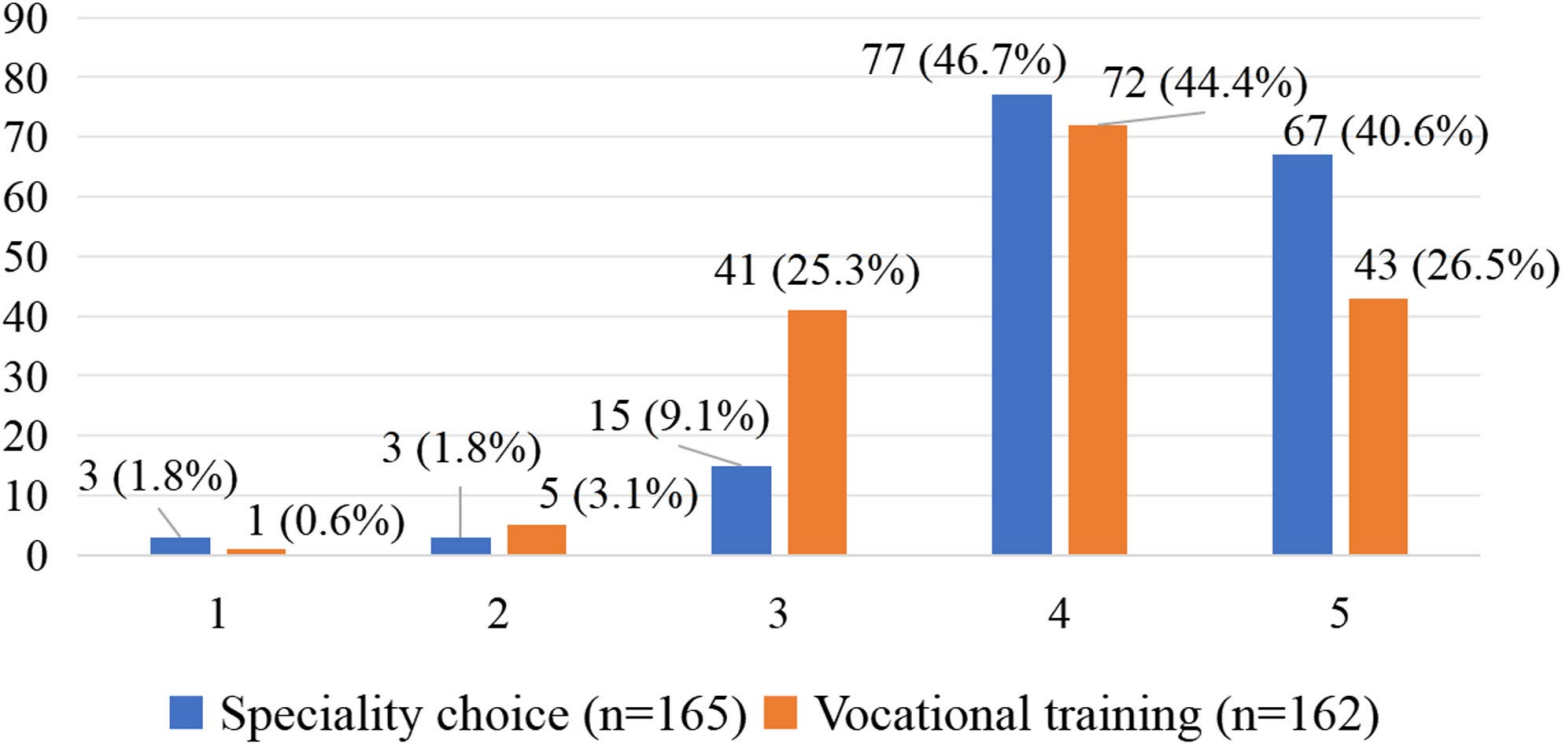
“If you could choose a specialty again, how likely is it that you would choose family medicine again?” and satisfaction with the vocational training

When asked whether they would choose family medicine again, nearly 90% of respondents selected values of 4 or 5 on a 5-point Likert scale. Overall, 70.9% of residents reported satisfaction with their vocational training, rating it 4 or 5 on a 5-point Likert scale (Fig. 2).

Residents were asked what they would suggest to improve the vocational training program and the most frequently suggested areas were management competencies (*n* = 16), increased time spent in family practice (*n* = 15), and expanded clinical practice (*n* = 15). After specialization, 52.9% of respondents (*n* = 157) plan to work as GP in their own practice, 37% as an employed GP, 12.1% would like to work in primary care but not in a family practice, 7% plan to work in healthcare but not in primary care, while 1.9% plan to leave healthcare. 68.5% of respondents plan to obtain additional licenses. Almost 75% of residents plan to take an additional specialization exam (Table 2).

**Table 2:** Resident doctors’ plan to obtain additional licenses or specializations

	Additional license (*n* = 162)		Additional specialization exam	
1.	Diabetology	62 (38.3%)	Occupational medicine	67 (40.6%)
2.	Hypertonology	39 (24.1%)	Psychotherapy	31 (18.8%)
3.	Palliative medicine	26 (16%)	Sports medicine	23 (13.9%)
			Other	41 (24.9%)
	Don’t plan	51 (31.5%)	Don’t plan	42 (25.5%)

Male gender was significantly associated with higher levels of depersonalization (*p* < 0.001; *r* = 0.300) and hopelessness (*p* = 0.025; *r* = 0.177), while no other significant associations were observed between demographic variables and mental health outcomes.

Significant associations were found between emotional exhaustion (*p* = 0.006; *r* = -0.240), hopelessness (*p* < 0.001; *r* = -0.243 ), and satisfaction with career choice. Satisfaction with vocational training was significantly associated with higher levels of emotional exhaustion (*p* < 0.001; *r* = -0.242), depersonalization (*p* < 0.001; *r* = -0.287), and reduced personal accomplishment (*p* < 0.001; *r* = 0.243).

We did not find significant correlations between intention to obtain additional license and burnout (emotional exhaustion: *p* = 0.266; depersonalization: *p* = 0.159; reduced personal accomplishment: *p* = 0.115), depression (*p* = 0.370), or hopelessness (*p* = 0.516).

We did not find significant correlations between intention to pursue an additional specialization exam and burnout (emotional exhaustion: *p* = 0.089; depersonalization: *p* = 0.210; reduced personal accomplishment: *p* = 0.276), depression (*p* = 0.407), or hopelessness (*p* = 0.230).

## Discussion

In this national cross-sectional study, Hungarian GP residents reported high levels of satisfaction with their career choice and vocational training, despite a considerable prevalence of burnout, particularly reduced personal accomplishment. High satisfaction with career choice may coexist with impaired mental health, indicating that positive professional identity alone does not protect against burnout. Severe depression and hopelessness affected a smaller, but clinically relevant proportion of residents. Importantly, emotional exhaustion dimension of burnout and hopelessness were significantly associated with lower satisfaction with career choice, while lower satisfaction with vocational training significantly correlated with all the three dimensions of burnout. These findings highlight a complex coexistence of professional commitment and impaired mental well-being among GP residents. The predominance of reduced personal accomplishment suggests that GP residents may experience diminished professional efficacy rather than emotional detachment or exhaustion. This pattern may be related to uncertainties regarding role expectations, workload and preparedness for independent practice.

Mental health problems occur frequently among Hungarian residents. The point prevalence of depression was 21.1% and severe depression was found in 4.8%, which are higher proportions than those reported in the general population in Hungary. In our sample depression was found less frequent as among residents from the most european countries but more frequent as in the general population [15, 21–26]. Male participants exhibited higher levels of burnout in the depersonalization dimension and hopelessness compared to their female counterparts. This finding contrasts with the majority of the existing literature, which generally reports a higher prevalence and greater severity of burnout, particularly emotional exhaustion, among female healthcare professionals [42]. Further investigation is warranted to better understand the mechanisms contributing to these gender-specific patterns.

Career choice and vocational training satisfaction are multifactorial issues. Factors related to undergraduate and vocational training, the political and media environment, personal role models, and work-life balance are also important influences. Notably, most residents plan to work in primary care and family practice, making their request for more time in family practice particularly relevant and forward-looking. The majority plan to work in their own practice, and high-level management competencies are necessary to succeed in this. Residents’ suggestions for improving vocational training—particularly the need for stronger management competencies and increased time spent in family practice—reflect gaps that are not fully addressed in the current training structure. Our results are consistent with international studies showing that residents are generally satisfied with their clinical competencies, while uncertainty and instability persist regarding management and practice leadership competencies [9].

Although licenses can be obtained not just by general practitioners and it does not exist a register about licensed general practitioners, but the total number of licenses can serve as a guide for comparison with the residents’ plans. The total number of the registered licenses from 2014 are 1071. Based on this the licensed GPs surely do not reach 20% in Hungary. In our study was found a much more higher proportion. Probably it will not be fully realized but it may indicate greater motivation to professional development. The popularity of the licenses among residents is the same as in the previous years, diabetology, hypertonology and palliative care [10].

A higher proportion of residents plan to take additional specialization exam than the proportion of current GPs who have additional specialization (74.5% vs. 52%). It is important to mention that a significant proportion of active practicing general practitioners with more specializations obtained their other specialty examinations before starting their career in the field of family medicine. This may explain why it is a relevant difference between the residents’ preferences (psychotherapy, sports medicine) and the actuell second specializations (e.g. internal medicine, anaesthesiology). The popularity of occupational medicine in both groups can be explained by the fact that it can be a part-time profession as a general practitioner.

The aim of the current study was to describe the current situation and not to directly examine solutions, but we consider it is important to briefly summarize good practices based on the available literature data that could be implemented in the Hungarian system. Targeted interventions in Hungarian family medicine residency program could improve residents’ mental health and enhance their training experiences. It is widely recognized that both individually focused and organizationally focused interventions can play a significant role in improving well-being and reducing burnout among healthcare professionals [43]. Different mindfulness-based practices or yoga can effectively reduce anxiety, depression, and all three dimensions of burnout [44, 45]. Bálint groups, which are increasingly accessible to Hungarian GP residents, could have a substantial positive impact on residents’ mental health and interpersonal skills [16, 46]. While individual-level interventions such as mindfulness-based programs and Bálint groups may support coping strategies, organizational-level changes addressing workload and administrative burden are likely to have a broader and more sustainable impact. Reducing workload through organizational interventions and the reevaluation of responsibilities could positively impact healthcare professionals’ mental health [47]. Therefore, new strategies in the Hungarian primary healthcare system, such as the creation of GP clusters and the integration of Advanced practice nurses, could not only improve the quality of the services but also protect GPs’ mental health.

### Limitations of the study

Self-selection bias could be a limitation of the study. Although we can assume that the characteristics of the participant group of residents and the non-participant group of residents are not different. The gender ratio supports our assumption: 62.1% (106/161) in the participant group and 66.67% (18/27) in the non-participant group were female. Participation during resident meetings may have introduced overrepresentation of more motivated and more engaged residents. The cross-sectional design does not allow causal inferences, and the use of self-reported measures may be subject to reporting bias. Finally, the study was conducted in a single national context, which may limit the generalizability of the findings.

Future longitudinal studies are needed to explore causal pathways between vocational training experiences, mental health and long-term career trajectories among GP residents.

## Conclusions

Most Hungarian general practitioner residents are satisfied with their career choice and vocational training, despite a considerable prevalence of burnout and other mental health problems. Satisfaction with vocational training was closely associated with residents’ well-being and future career perspectives. These findings underline the importance of addressing both educational and organizational factors during residency training. Supporting GP residents’ mental health may contribute to retention in primary care and help strengthen the long-term sustainability of the primary healthcare workforce.

## Supplementary Information

Supplementary Material 1.

## Data Availability

The datasets used and/or analyzed during the current study are available from the corresponding author on reasonable request.

## Abbreviations

ANOVA: Analysis of variance
BDI-9: Shortened Beck Depression Inventory
CI: Confidence interval
FM: Family medicine
GP: General practitioner
HS-4: Short hopelessness scale
IBM-SPSS-24: International Business Machines Corporation - Statistical Package for the Social Sciences - version 24
MBI: Maslach Burnout Inventory
MBI-D: Maslach Burnout Inventory - Depersonalization
MBI-E: Maslach Burnout Inventory - Emotional exhaustion
MBI-I: Maslach Burnout Inventory - Impaired personal accomplishment
*P*-value: Probability value (used for statistical significance)
SD: Standard deviation
