# Supplementary material for: General practitioner residents’ mental health and satisfaction during their vocational training
Source: BMC Prim Care. 2026 Apr 14;27:204. doi: 10.1186/s12875-026-03317-2 (PMC13196140; doi:10.1186/s12875-026-03317-2)
Supplement: Supplementary file 1 — Supplementary Material 1. [file 12875_2026_3317_MOESM1_ESM.docx]

# ****Attitudes and Motivations Related to Professional Commitment and Specialty Choice Among Family Medicine Residents and Trainees****

Dear Colleague,

We kindly ask you to support our research by completing this questionnaire, which examines the attitudes and motivations of family medicine residents and trainees regarding specialty choice and professional commitment. Participation is voluntary and anonymous. Your personal data will be handled exclusively by the persons responsible for the study and solely for research purposes. The data will not be used for any other purpose nor disclosed to third parties.

If you have any questions, please contact:
**Contact person:** Dr. András Mohos (mohosandris@gmail.com)

Thank you for your cooperation!

## Personal Data

At which university are you completing your family medicine specialty training?
 University of Debrecen University of Pécs Semmelweis University University of Szeged

Age: ____ years
Gender: Male Female

Marital status: Single In a relationship (separate household)
 Cohabiting (same household) Married Divorced Widowed

Do you have children? Yes, number: ______ No

Year of obtaining your medical degree: ______
Year of starting family medicine specialty training: ______
What stage of your specialty training are you currently in?
First year Second year Third year Immediately before board examination

Do you have another specialist qualification?
 Yes, namely: ____________________ No

Before starting family medicine specialty training, did you work in another healthcare/social field?
 Yes, what and for how long? ____________________ No
Before starting family medicine specialty training, did you work in a NON-healthcare/social field?
 Yes, what and for how long? ____________________ No

Where did you grow up?
 Capital city Large city (>20,000 inhabitants)
 Small town (<20,000 inhabitants) Village

Where do you currently live?
 Capital city Large city Small town Village

Where is the general practice located where you complete your training?
County: _____________
 Capital city Large city Small town Village

Where do you plan to live long-term in the future? Hungary Abroad

Where do you plan to live long-term in the future? (Multiple answers possible)
 Capital city Large city Small town Village

Where do you plan to work long-term in the future? (Multiple answers possible)
 Capital city Large city Small town Village

# Choice of speciality and workplace; Role of vocational training

When did you decide to choose a medical career?
 Childhood dream During primary school During secondary school After secondary school

When did you decide to choose family medicine?
 Childhood dream During primary school During secondary school During medical university Immediately after graduation Later

At the time of specialty choice, family medicine was my first choice: Yes No

Which specialty/specialties ranked ahead of family medicine? ___________________

If you could choose again, how likely would you be to choose family medicine again?
 Definitely not Probably not Unsure Probably yes Definitely yes

Where would you most likely work long-term?
 Own private practice (self-employed)
 Municipality-operated practice (healthcare service employment status)
 As an employed physician in another GP’s practice
 In primary care without a permanent practice (on-call/substitution)
 In healthcare outside primary care
 Outside healthcare
 I do not know yet

If you would like to own a practice, where would you prefer to work?
 Capital city Large city Small town Village Abroad

How likely do you consider it that you will work long-term in your own practice?
(1 = definitely not, 5 = definitely yes) 1 2 3 4 5

How likely do you consider it that you will work long-term in a rural (small town/village) practice?
(1 = definitely not, 5 = definitely yes) 1 2 3 4 5

How likely do you consider it that you will work long-term in a mixed (adult and pediatric) practice?
(1 = definitely not, 5 = definitely yes) 1 2 3 4 5

If unlikely (score 1–3), what is the reason? _____________________________________________
________________________________________________________________________________

If you plan to own a practice, how long after obtaining your board certification would you plan to do so?
 Within 1–2 years 3–5 years 5–10 years More than 10 years I do not plan to buy a practice

Please describe the factors that make owning a practice attractive to you: ______________________________________________________________________________________________________________________________________________________________________________

Please describe the factors that make owning a practice unattractive to you: ______________________________________________________________________________________________________________________________________________________________________________

To what extent does family medicine training prepare you for working in your own practice?
 Not at all To a small extent To some extent To a great extent Completely

Overall, how satisfied are you with family medicine specialty training?
(1 = not at all, 5 = completely) 1 2 3 4 5

What specific suggestions do you have for improving family medicine specialty training? ______________________________________________________________________________________
______________________________________________________________________________________

After obtaining your family medicine board certification, do you plan further specialty training?
 Yes No

If yes, which of the following? Occupational medicine Internal medicine Emergency medicine Sports medicine Psychotherapy
 Other: _________________

Do you plan to obtain a license-based subspecialty qualification? Yes No

If yes, which of the following? Diabetology Hypertension care Palliative medicine

# Motivations for Choosing Family Medicine

Please indicate to what extent the following factors played a role in your choice of family medicine.
(1 = Not at all, 5 = Completely)

Work–life balance 1 2 3 4 5
Acceptable working conditions/working hours 1 2 3 4 5
Long-term financial prospects/salary 1 2 3 4 5
Career opportunities 1 2 3 4 5
Available training placements 1 2 3 4 5
Available job opportunities 1 2 3 4 5
Preparation for an academic career 1 2 3 4 5
Opportunity to work abroad 1 2 3 4 5
Family influence 1 2 3 4 5
Advice from others 1 2 3 4 5
It just happened this way 1 2 3 4 5
Influences during medical university 1 2 3 4 5
This is what truly interests me 1 2 3 4 5
Professional challenges 1 2 3 4 5
Innovation and development opportunities 1 2 3 4 5
Patient relationships 1 2 3 4 5
Professional prestige within the medical field 1 2 3 4 5
Social prestige 1 2 3 4 5

# Expected Income and Specialty/Workplace Choice

To what extent did expected income influence your choice of specialty/workplace?
(1 = no influence, 10 = very strong influence)
 1 2 3 4 5 6 7 8 9 10

Would you decide against a particular specialty/workplace if it involved lower expected income?
 Definitely not Probably not Probably yes Definitely yes

What would you consider an ideal net monthly income (HUF) for:
 Resident physician: __________
 Non-GP specialist: __________
 General practitioner: __________

(*Net income = amount received after all taxes and deductions; please consider all income sources.)

Please estimate the monthly net income (HUF) of the following full-time physicians and indicate how certain you are (1 = very uncertain, 4 = very certain):

GP resident – Large city: _________ 1 2 3 4
GP resident – Small town/rural: _________ 1 2 3 4
GP specialist – Large city: _________ 1 2 3 4
GP specialist – Small town/rural: _________ 1 2 3 4

Your current total monthly net income (HUF):
Below 500,000 501–600,000 601–700,000 701–800,000 801–900,000 901,000–1,000,000 1–1.5 million More than 1.5 million

Do you receive a resident scholarship? Yes No

If yes, which one? Markusovszky Lajos Scholarship Méhes Károly Scholarship
 Gábor Aurél Scholarship Shortage specialty scholarship Other

# Professional Recognition and System Evaluation

How would you rate the overall moral recognition of family physicians today? 1 (Poor) – 5 (Excellent)
 1 2 3 4 5

How would you rate the moral recognition of family physicians by other specialists? 1 (Poor) – 5 (Excellent)
 1 2 3 4 5

Please rate (1 = very poor, 5 = very good):
The current state of Hungarian healthcare 1 2 3 4 5
The state of Hungarian primary care/family medicine 1 2 3 4 5
Your current situation as an employee in healthcare 1 2 3 4 5

Please rate how you expect the following to change in the next five years (1 = much worse, 5 = much better):
Hungarian healthcare 1 2 3 4 5
Hungarian primary care/family medicine 1 2 3 4 5
Your situation as an employee in healthcare 1 2 3 4 5

Do you plan to work abroad within the next 3–5 years? Yes No
If yes, why? ___________________________________________________________________

# Informal Payments

Overall, what is your opinion about the system of informal payments (“gratitude payments”)?
 Completely reject Occasionally accept regardless of amount Occasionally accept below 8,000 HUF Always accept regardless of amount
 Always accept below 8,000 HUF

**Please rate the following statements from 0 to 6!
(**0: never 1: a few times a year 2: once a month 3: a few times a month
 4: once a week 5: a few times a week 6: every day)

I feel emotionally drained because of my work. _____

I feel completely worn out at the end of the day. _____

I feel tired when I get up in the morning and have to face another day at work. _____

I can easily understand how my colleagues/patients feel about certain things. _____

I feel I treat some people as if they were impersonal objects. _____

It is stressful for me to work with people all day. _____

I deal very effectively with the problems of my colleagues/patients. _____

I feel burned out from my work. _____

I feel that through my work I positively influence other people’s lives. _____

Since I started working here, I have become much more insensitive. _____

I am worried that my work is making me emotionally hard. _____

I feel very energetic. _____

My work irritates me. _____

I feel I work too hard. _____

I do not really care what happens to some of my colleagues/patients. _____

Working directly with people is too stressful for me. _____

I can easily create a calm atmosphere for my colleagues/patients/clients. _____

Working with people energizes me. _____

I have accomplished many worthwhile things in my job. _____

I feel like I’m at the end of my rope. _____

In my work, I handle emotional problems very calmly. _____

I feel that my colleagues/patients blame me for some of their problems. _____

**Please rate the following statements!**
(1: Not true at all 2: Partly true 3: Largely true 4: Completely true)

I see my future as dark 1 2 3 4

Things will never turn out the way I want them to 1 2 3 4

There is no point in really striving for something I want, because I probably won’t achieve it anyway

1 2 3 4

I see my future as hopeless and feel that my situation will not change 1 2 3 4

xxxxxxxxxxxxxxxxxxxxxxxxxxxxxxxxxxxxxxxxxxxxxxxxxxxxxxxxxxxxxxxxxxxxxxxxxxxxxxxxxxxxxxx

(1: Not characteristic at all 2: Slightly characteristic 3: Characteristic 4: Completely characteristic)

I have lost all interest in other people 1 2 3 4

I can no longer make decisions about anything 1 2 3 4

I wake up several hours earlier than usual and cannot fall back asleep 1 2 3 4

I am too tired to do anything 1 2 3 4

I worry so much about physical complaints that I cannot think about anything else 1 2 3 4

I am unable to carry out any kind of work 1 2 3 4

I see the future as hopeless and feel that my situation will not improve 1 2 3 4

I am dissatisfied with everything or indifferent 1 2 3 4

I constantly blame myself 1 2 3 4

*If you feel that you, your relative, friend or colleague need immediate assistance, support is available 24 hours a day through the following online platforms or telephone numbers. The phone numbers listed below can be called free of charge from any network. Hungarian Association of Mental Health First Aid Services (LESZ): 116-123 (available 24/7, free of charge)
E-mail: sos116123@gmail.com (reply within 72 hours)*

**Thank you for completing the questionnaire!**
